# The Rise and Fall of Omicron BA.1 Variant as Seen in Wastewater Supports Epidemiological Model Predictions

**DOI:** 10.3390/v15091862

**Published:** 2023-08-31

**Authors:** Michal Liddor Naim, Yu Fu, Marilou Shagan, Itay Bar-Or, Robert Marks, Qun Sun, Rony Granek, Ariel Kushmaro

**Affiliations:** 1 Avram and Stella Goldstein-Goren Department of Biotechnology Engineering, Ben-Gurion University of the Negev, Beer-Sheva 84105, Israel; 2 Key Laboratory of Bio-Resources and Eco-Environment of the Ministry of Education, College of Life Sciences, Sichuan University, Chengdu 610064, China; 3 Central Virology Laboratory, Public Health Services, Ministry of Health, Chaim Sheba Medical Center, Ramat Gan 5262000, Israel; 4 The Ilse Katz Center for Nanoscale Science and Technology, Ben-Gurion University of the Negev, Beer-Sheva 84105, Israel; 5 School of Sustainability and Climate Change, Ben-Gurion University of the Negev, Beer-Sheva 84105, Israel

**Keywords:** SARS-CoV-2, Omicron, wastewater-based epidemiology, RT-qPCR, SIR model, SIRS model

## Abstract

The COVID-19 pandemic caused by the SARS-CoV-2 virus has inflicted significant mortality and morbidity worldwide. Continuous virus mutations have led to the emergence of new variants. The Omicron BA.1 sub-lineage prevailed as the dominant variant globally at the beginning of 2022 but was subsequently replaced by BA.2 in numerous countries. Wastewater-based epidemiology (WBE) offers an efficient tool for capturing viral shedding from infected individuals, enabling early detection of potential pandemic outbreaks without relying solely on community cooperation and clinical testing resources. This study integrated RT-qPCR assays for detecting general SARS-CoV-2 and its variants levels in wastewater into a modified triple susceptible-infected-recovered-susceptible (SIRS) model. The emergence of the Omicron BA.1 variant was observed, replacing the presence of its predecessor, the Delta variant. Comparative analysis between the wastewater data and the modified SIRS model effectively described the BA.1 and subsequent BA.2 waves, with the decline of the Delta variant aligning with its diminished presence below the detection threshold in wastewater. This study demonstrates the potential of WBE as a valuable tool for future pandemics. Furthermore, by analyzing the sensitivity of different variants to model parameters, we are able to deduce real-life values of cross-variant immunity probabilities, emphasizing the asymmetry in their strength.

## 1. Introduction

The COVID-19 pandemic due to SARS-CoV-2 is responsible for huge mortality and severe morbidity, and the virus is still circulating globally [1,2]. Meanwhile, the SARS-CoV-2 virus is undergoing mutations resulting in the continuous emergence of new variants [3,4,5]. The Omicron (B.1.1.529) variant was first detected in South Africa in November 2021 and had undergone significant mutations compared to its previous variants, which were considered to have increased virulence and pathogenicity. Due to its numerous mutations and transmissibility rate, it could spread rapidly [6,7,8]. Globally, the BA.1 sub-lineage emerged as the predominant variant; however, it was gradually supplanted by BA.2 in numerous countries [9,10]. Analysis of SARS-CoV-2-positive sample sequences in Israel revealed the prevailing dominance of the BA.2 variant between March 2022 and June 2022 [11]. Until now, Omicron and sister lineages are currently the dominant variants circulating globally, accounting for >98% of viral sequences shared on GISAID after February 2022 [12].

Wastewater-based epidemiology (WBE) captures viral shedding from infected individuals, irrespective of clinical presentation; it can be used as a convenient tool that provides virus prevalence in a population [13,14,15,16,17,18,19]. The utilization of such a tool allows for the early detection and warning of potential pandemic outbreaks, circumventing the reliance on community cooperation and clinical testing resources. Previous studies conducted in our lab systematically monitored SARS-CoV-2 variant occurrence in wastewater by employing the reverse transcriptase quantitative polymerase chain reaction (RT-qPCR) method [17,18,20]. This virus surveillance facilitated the development of a double susceptible-infected-recovered (SIR) model specifically tailored for the Delta and Omicron variants [20].

In light of the widespread transmission of the Omicron virus of concern across the globe, the aim of this research is to enable a quick assessment of the spread of SARS-CoV-2 variants via WBE [20] and, in parallel, use SIR model for predicting the possible future behavior of the variant, which can be verified by RT-qPCR in return. Therefore, WBE data can be used to optimize prediction modeling for infectious agents, including the virus spread in the population. This study aims to integrate RT-qPCR assays for detecting general SARS-CoV-2 and the levels of its variants in wastewater into a modified triple susceptible-infected-recovered-susceptible (SIRS) model.

## 2. Materials and Methods

### 2.1. Wastewater Samples

Composite 24 h influent wastewater samples were obtained from the Beer Sheva municipality wastewater treatment plant in Israel. The samples were collected twice a week using an autosampler from December 2021 to July 2022. Samples were transported to the lab under chilled conditions and stored at 4 °C until further processing.

### 2.2. RNA Extraction

RNA was extracted from the wastewater samples using a Zymo Environ Water RNA kit (Zymo Research R2042) according to the manufacturer protocol, including the viral enrichment step as described previously by Yaniv et al. (2022) [20]. The MS2 phage, present in undetectable levels in Israeli wastewater, served as an external control during the experiment by spiking the samples with 105 copies. The RNA samples were eluted using 35 μL of RNase-free water and preserved at a temperature of −80 °C.

### 2.3. RT-qPCR

Taq-Man RT-qPCRs assays for detecting and quantifying SARS-CoV-2 in wastewater samples were performed as previously described [17,18,20] using Applied Biosystems Thermocycler (Thermo Scientific). The primer and probes used by Yaniv et al. (2022) [17,18,20] and listed in Table 1. Following the manufacturer’s protocol, the assay used One Step PrimeScript III RT-qPCR mix RR600 (Takara, Japan). The reaction mixture contains 0.5 μM primers, 0.2 μM probes, and added 5 μL of RNA sample to the total volume of 20 μL. RNA copy number per liter of wastewater was calculated using calibration curves and Equation S1 presented in the supplementary and following Yaniv et al. (2022) [20].

## 3. Results

### 3.1. Wastewater Portrait of SARS-CoV-2

**Figure 1:**
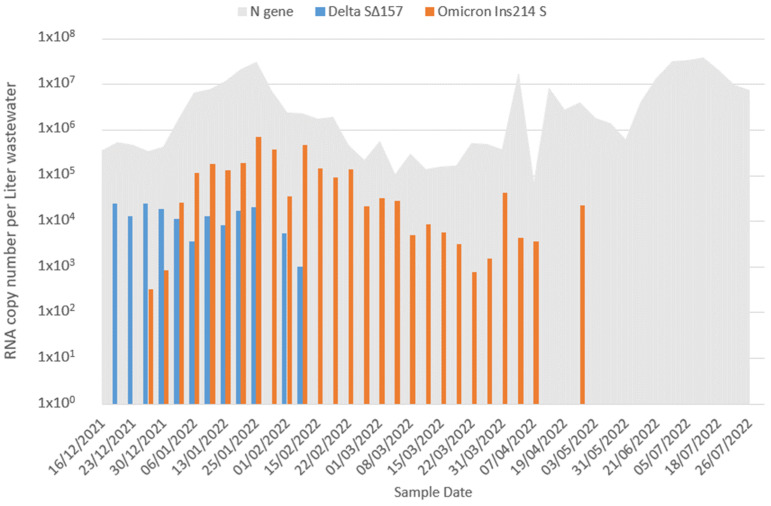
RT-qPCR of SARS-CoV-2, Delta, and Omicron BA.1 variants in Beer Sheva wastewater from mid-December 2021 to July 2022. The gray area illustrates the overall SARS-CoV-2 variants using N gene sets. The blue columns indicate the detection of the Delta variant using the SΔ157 set, while the orange columns represent the detection of the Omicron variant of concern using the Ins214S set.

The wastewater of Beer Sheva, Israel’s fourth largest city, has been continuously monitored since the beginning of the COVID-19 pandemic, as detailed in previous publications [17,18,20]. The detection of general SARS-CoV-2 levels was carried out utilizing the CDC’s N2 set, while the specific identification of the Delta and Omicron BA.1 variants was achieved through the utilization of the Delta S∆157 and Omicron Ins214 S sets developed by Yaniv et al. (2021, 2022) [17,20]. This study monitored general SARS-CoV-2 and the shifting of variants from the end of December 2021 until July 2022 (Figure 1). Delta variant copy numbers declined and was undetectable after the beginning of February 2022. From the beginning of January 2022, Omicron BA.1 was the dominant variant. The peak of Omicron BA.1 was reached at the end of January until the beginning of February 2022. Omicron BA.1 decreased to an undetectable level in the wastewater after April 2022. The remaining high level of overall SARS-CoV-2 (through N gene monitoring) is probably due to the emerge of the Omicron BA.2 variant in Israel during February 2022 and followed by BA.4 and BA.5 in the mid of April [11], which is undetectable by the Omicron Ins214 S probe that was used in this study (Figure S1) [20]. The WBE data presented in Figure 1 may help develop and optimize prediction modeling for infectious agents, including viruses spread in the population.

### 3.2. Remodeling

Soon after the Omicron BA.1 outbreak, Omicron BA.2 emerged [11]. It is essential to include it too, as it has a larger basic reproduction number (*R*_0_) than BA.1 while inducing strong protection against BA.1 and similar protection as BA.1 against Delta. We, therefore, present here a triple susceptible-infected-recovered-susceptible (SIRS) model [22,23] that accounts for an (exponentially decaying) time-dependent waning immunity and cross-variant immunization [20].

We define the following time-dependent variables:

1. $f_{D}$, $f_{O}$, and $f_{B}$ are the fractions of actively infected populations in Delta, Omicron BA.1, and Omicron BA.2, respectively.
2. $s_{D}$, $s_{O}$, and $s_{B}$ are the effective fractions of susceptible populations to Delta, Omicron BA.1, and Omicron BA.2 infections, respectively, henceforth “susceptibilities”. These variables present an average over the diverse immunity presented in the population, although, in the original SIR model, they simply present the fraction of population that is neither actively infected nor recovered.
3. $r_{D}$, $r_{O}$, and $r_{B}$ are the fractions of recovered population from Delta, Omicron BA.1, and Omicron BA.2, respectively. The contribution of recovered individuals from previous outbreaks is accounted for in the initial conditions.

In addition, we use the following (time-independent) parameters:

1. $\tau_{D}$, $\tau_{O}$, and $\tau_{B}$ are the infection time-periods of Delta, Omicron BA.1, and Omicron BA.2, respectively.
2. $R_{0}^{\left(D\right)}$, $R_{0}^{\left(O\right)}$, and $R_{0}^{\left(B\right)}$ are the basic reproduction numbers of Delta, Omicron BA.1, and Omicron BA.2, respectively.
3. $\tau_{rD}$, $\tau_{rO}$, and $\tau_{rB}$ are the corresponding characteristic waning-immunity times, based on exponential decay of the immunity.

The model equations are as follows:

(1) $$\frac{d}{dt}f_{D}=\frac{R_{0}^{\left(D\right)}}{\tau_{D}}s_{D}f_{D}-\frac{f_{D}}{\tau_{D}}$$

(2) $$\frac{d}{dt}f_{O}=\frac{R_{0}^{\left(O\right)}}{\tau_{O}}s_{O}f_{O}-\frac{f_{O}}{\tau_{O}}$$

(3) $$\frac{d}{dt}f_{B}=\frac{R_{0}^{\left(B\right)}}{\tau_{B}}s_{B}f_{B}-\frac{f_{B}}{\tau_{B}}$$

(4) $$\frac{d}{dt}r_{D}=\frac{f_{D}}{\tau_{D}}-\frac{r_{D}}{\tau_{rD}}$$

(5) $$\frac{d}{dt}r_{O}=\frac{f_{O}}{\tau_{O}}-\frac{r_{O}}{\tau_{rO}}$$

(6) $$\frac{d}{dt}r_{B}=\frac{f_{B}}{\tau_{B}}-\frac{r_{B}}{\tau_{rB}}$$

where $s_{D}$, $s_{O}$, and $s_{B}$ take the following expressions, accounting for cross-variant immunization,

(7) $$s_{D}=1-\left(f_{D}+r_{D}\right)-q_{DO}r_{O}-q_{DB}r_{B}$$

(8) $$s_{O}=1-\left(f_{O}+r_{O}\right)-q_{OD}r_{D}-q_{OB}r_{B}$$

(9) $$s_{B}=1-\left(f_{B}+r_{B}\right)-q_{BD}r_{D}-q_{BO}r_{O}$$

In Equations (1)–(3), the first term on the right-hand-side (RHS) of each equation represents an infection rate while the second term accounts for the recovery rate. In Equations (4)–(6), the first term on the RHS in each equation is the same recovery rate, while the second term is a waning-immunity rate.

In Equations (7)–(9), $q_{DO}$ represents the relative mean protection against Delta infection that a newly recovered individual from Omicron BA.1 gained, and similarly $q_{OD}$ represents the relative mean protection against Omicron BA.1 infection that a newly recovered individual from Delta gained; $q_{BO},\;q_{OB}$, $q_{DB}$, and $q_{BD}$ have a corresponding meaning.

To minimize free-parameters, and due to the similarity between Omicron BA.1 and Omicron BA.2, we used $q_{DO}=q_{DB}$, $q_{OD}=q_{BD}$. Asymmetric cross-immunization, supported by antibody immunology studies [24,25], implies $q_{DO}<q_{OD}$, i.e., the protection against Omicron BA.1 due to Delta past infection is higher than the protection against Delta due to Omicron BA.1 past infection. However, these antibody immunity assays do not account for the full complexity of the immune system, such as the T-cell immunity, and so are an underestimate of the immunity against Delta due to Omicron BA.1 past infection. Hence, the estimated ratio $q_{DO}/q_{OD}=0.25$ used in our previous publication [20], which stems from these immunity assays, is an underestimate, which we correct here to be $q_{DO}/q_{OD}=$ 0.6 to fit better the wastewater data. (In the SI of Yaniv et al. (2022) [20] we considered also $q_{DO}/q_{OD}=$0.5, closer to the ratio used here.)

To account for immunity gained from past pandemic waves and vaccination, the initial recovered fraction of Delta is set to a non-zero value, thereby determining the initial susceptibilities (t=0) associated with the three variants.

In addition, we use the following parameter values that fall within the acceptable range of known values to roughly fit the observed data:

1. Basic reproduction numbers
  $$R_{0}^{\left(D\right)}=3.7$$
  $$R_{0}^{\left(O\right)}=7.3$$
  $$R_{0}^{\left(B\right)}=1.5\times R_{0}^{\left(O\right)}=10.95$$
2. Infection periods
  $$\tau_{D}=11\;(\mathrm{days})$$
  $$\tau_{O}=7\;(\mathrm{days})$$
  $$\tau_{B}=7\;(\mathrm{days})$$
3. Characteristic waning-immunity times
  $$\tau_{rD}=550\;(\mathrm{days})$$
  $$\tau_{rO}{=\tau }_{rB}=400\;(\mathrm{days})$$
4. Cross immunity probabilities
  $$q_{OD}=0.66,\;q_{DO}=0.6\times q_{OD}=0.396$$
  $$q_{BD}=0.66,\;q_{DB}=0.6\times q_{BD}=0.396$$
  $$q_{OB}=0.8,\;q_{BO}=1.0875\times q_{OB}=0.87$$

The following initial conditions are taken: $f_{D}\left(0\right)=6.641\times 10^{-3}$, $f_{0}\left(0\right)=3.3375\times 10^{-5}$, $r_{D}\left(0\right)=0.724$, $r_{O}\left(0\right)=r_{B}\left(0\right)=0$. The entrance of BA.2 is modeled as a (Dirac-delta function) source rate at day 34 with value $10^{-5}$ (${day}^{-1}$), introduced on the RHS of Equation (3).

**Figure 2:**
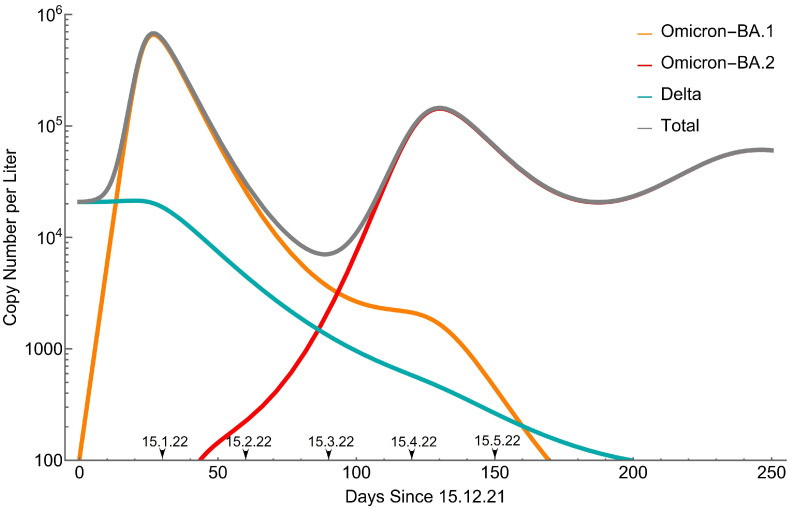
SARS-CoV-2 variants copy number from the model, scaled to the observed wastewater concentrations (Figure 1).

Equations (1)–(9) form a set of non-linear differential equations and are therefore solved numerically. The results for the active infections ($f_{D}$, $f_{O}$, and $f_{B}$), scaled to the observed wastewater peak concentration of the Omicron BA.1 wave, are shown in Figure 2.

Compared to the wastewater data (Figure 1), the BA.1 wave is well described. The subsequent BA.2 wave, roughly 4+ months later, is consistent with the total wastewater viral count and MOH infection data [26]. Soon after BA.1 peaked, there was a strong drop of Delta, consistent with it going below the detection threshold. This drop continues even after BA.1 dropped to low values, since the BA.2 wave keeps it down. In absolute terms, according to the model Delta is not eradicated completely, but it is kept extremely low.

The BA.2 wave keeps BA.1 dropping too, but not at a constant pace, and we can observe a “shoulder” of it roughly when BA.2 peaks, reminiscent of the “shoulder” in wastewater data (end March–early April). In the absence of BA.2, we would have had a second BA.1 wave coming due to the BA.1 waning immunity, and the “shoulder” is simply the signature left from this hypothetical wave before the BA.2 wave suppresses it. Note that, surprisingly, BA.1 is not detected in wastewater for the subsequent few weeks in April and emerges briefly at end-April (Figure 1), which is clearly not predicted by the model.

In Figures S2–S4, we perform sensitivity analysis to the variation of a few parameters: (i) $R_{0}^{\left(B\right)}$ (Figure S2), ranging from 30% to 60% above $R_{0}^{\left(0\right)}$ [10], showing relatively weak sensitivity of all variants. (ii) $q_{DO}$ and $q_{DB}$ (Figure S3), ranging from 40% to 70% of $q_{OD}$ and $q_{BD}$, respectively, thus describing strong asymmetric cross-immunities between the pairs BA.1 and Delta and BA.2 and Delta. The analysis shows strong sensitivity of the faith of Delta but very weak sensitivity of the two other variants. (iii) $q_{BO}$ (Figure S3), ranging from just 117.5% to 91% of $q_{OB}$, thus describing slight asymmetric cross-immunities between BA.1 and BA.2. The analysis shows strong effects on BA.1 and BA.2 and weaker effects on Delta. In particular, the timing of the BA.2 wave is very sensitive to this value, which in turn influences the appearance of the shoulder/peak in BA.1.

The presented model is based on homogeneous infection spreading model and cannot describe spatial (geographic) non-uniformity [27,28,29,30]. We suspect that the brief (single detection) emergence of BA.1 in end-April results from a late infection of one of the city neighborhoods. Unfortunately, the data for different neighborhoods are unavailable to us, so we cannot confirm this hypothesis. Nevertheless, this suggests that detection at multiple locations, even within the same city, could give insight into the spreading pattern of disease [31].

## 4. Discussion

In this study, we are demonstrating the integration of RT-qPCR assays for detecting general SARS-CoV-2 and its Delta and Omicron BA.1 variant levels in wastewater into a modified triple susceptible-infected-recovered-susceptible (SIRS) model. Monitoring of Israel’s 4th largest city (Beer Sheva) was performed through wastewater analysis to determine SARS-CoV-2 levels in wastewater. With escalated global infections, the Omicron BA.1 variant appeared in the wastewater and eradicated the presence of its predecessor, the Delta variant (Figure 1). We see a situation similar to the one we describe in other parts of the world as the Omicron BA.1 variant spreads [32,33,34]. Using the wastewater direct RT-qPCR variant detection data (Figure 1) and the modified SIRS model (Figure 2), the BA.1 wave is well-described. The subsequent BA.2 wave, roughly 4+ months later, is consistent with the total wastewater viral count. Soon after BA.1 peaked, there was a strong drop of the Delta variant, consistent with it going below the detection threshold in wastewater. This drop continues even after BA.1 drops to low values since the BA.2 wave keeps it down. In absolute terms, according to the model, Delta is not eradicated completely but is kept extremely low.

This study demonstrated that integrating direct RT-qPCR results data from wastewater surveillance, can help optimize prediction modeling for infectious agents, including virus spread in the population, and may be useful for future pandemics. Due to the high sensitivity of the different variants to some of the model parameters (as seen in the SI, Figures S2–S4), we were able to roughly deduce real-life values of cross-variant immunity probabilities in terms of the strength of their asymmetry. Specifically, we suggest that the protection against Delta due to an Omicron BA.1 past infection is *lower by* about 40% than the protection against Omicron BA.1 due to a Delta past infection, and that the protection against Omicron BA.2 due to an Omicron BA.1 past infection is *higher by* about 10% than the protection against Omicron BA.1 due to an Omicron BA.2 past infection. Increasing the number of variants detected simultaneously, the detection accuracy, and improving the model, in particular accounting for geographic inhomogeneities in the disease spread, can improve the estimates of model parameters. Preparation for future pandemics should include these issues.

## Figures and Tables

**Table 1:** List of primers and probes sequences used in this study.

Name	Target	Primer/Probe	Sequence (5′ ->3′)	Database Accession Number	Position	Reference
nCoV_N2-F	All Variants	Forward	TTACAAACATTGGCCGCAAA	NC_045512.2 ^†^	29,164–29,183	CDC
nCoV_N2-R		Reverse	GCGCGACATTCCGAAGAA		29,230–29,213	
nCoV_N2-P		Probe	FAM/ACAATTTGCCCCCAGCGCTTCAG/3IABkFQ		29,188–29,210	
F21989 *	Delta	Forward	GTTTATTACCACAAAAACAACAAAAG	EPI_ISL_1704637 ^‡^	21,964–21,989	[17]
R22083 *		Reverse	GGCTGAGAGACATATTCAAAAGTG		22,052–22,029	
S∆157		Probe	FAM/TGGATGGAA/ZEN/AGTGGAGTTTATTCTAGT/3IABkFQ		21,991–22,017	
F22083	Omicron	Forward	TTAAAATATATTCTAAGCACACGC	EPI_ISL_6794907 ^‡^	22,083–22,106	[20]
R22181		Reverse	CATTTCGCTGATTTTGGGGTCC		22,157–22,181	
Ins214 S		Probe	FAM/TATTATAGT/ZEN/CGTGAGCCAGAAGATCTCC/3IABkFQ		28,215–28,244	
MS2-TM2-F	MS2	Forward	TGCTCGCGGATACCCG	V00642 ^†^	3169–3184	[21]
MS2-TM2-R		Reverse	AACTTGCGTTCTCGAGCGAT		3229–3210	
MS2-TM2JOE		Probe	HEX/ACCTCGGGTTTCCGTCTTGCTCGT/3IABkFQ		3186–3209	

^†^ NCBI database; ^‡^ GISAID database; * Positions listed for each sequence are from the relevant accession number. Forward and Reverse primers names of Delta correspond to the nucleotides position on the original sequence, when aligned together with the variants of concern.

## Data Availability

Not applicable.

## Supplementary Materials

The following supporting information can be downloaded at: https://www.mdpi.com/article/10.3390/v15091862/s1, Equation S1: Equation for RNA copy number per liter of wastewater for raw samples calculation; Figure S1: Primer and probe illustration in Omicron Variants; Figure S2: Sensitivity analysis to the basic reproduction number of BA.2; Figure S3: Sensitivity analysis to the Delta—BA.1 and Delta—BA.2 pairs cross-immunity parameters; Figure S4: Sensitivity analysis to the BA.1—BA.2 pair.
