# Supplementary material for: The Rise and Fall of Omicron BA.1 Variant as Seen in Wastewater Supports Epidemiological Model Predictions
Source: Viruses. 2023 Aug 31;15(9):1862. doi: 10.3390/v15091862 (PMC10536904; doi:10.3390/v15091862)
Supplement: Supplementary file 1 [file viruses-15-01862-s001.zip › viruses-2514081-supplementary.pdf]

Supplementary Materials

The rise and fall of Omicron BA.1 variant as seen in wastewater supports epidemiological model predictions

Michal Liddor Naim [1,†], Yu Fu [1,2,†], Marilou Shagan [1], Itay Bar-Or [3], Robert Marks [1,4], Qun Sun [2], Rony Granek [1,4,*] and Ariel Kushmaro [1,4,5,*]

[1] Avram and Stella Goldstein-Goren Department of Biotechnology Engineering, Ben-Gurion University of the Negev, Beer-Sheva 84105, Israel
[2] Key Laboratory of Bio-Resources and Eco-Environment of the Ministry of Education, College of Life Sciences, Sichuan University, Chengdu 610064, China
[3] Central Virology Laboratory, Public Health Services, Ministry of Health, Chaim Sheba Medical Center, Ramat Gan 5262000, Israel
[4] The Ilse Katz Center for Nanoscale Science and Technology, Ben-Gurion University of the Negev, Beer Sheva 84105, Israel
[5] School of Sustainability and Climate Change, Ben-Gurion University of the Negev, Beer-Sheva 84105, Israel
* Correspondence: rgranek@bgu.ac.il (R.G.); arielkus@bgu.ac.il (A.K.)
† These authors contributed equally to this work.

Supplementary Materials

Calibration curves

In order to calculate the copy number in the RT-qPCR assay, calibration curves were performed on a known-positive dsDNA gene block as previously described [17,20]. insertion[15]. Copy number calculations were calculated using the following Equation. The equation for RNA copy number per Liter of wastewater for raw samples calculation

$$RNA\ copy\ number\ (\#/Liter\ of\ wastewater)$$
$$= \frac{10^{calibration\ curve} \bullet Elution\ volume}{RTqPCR\ reaction\ volume\ \bullet volume\ taken\ from\ wastewater\ for\ RNA\ extraction}$$

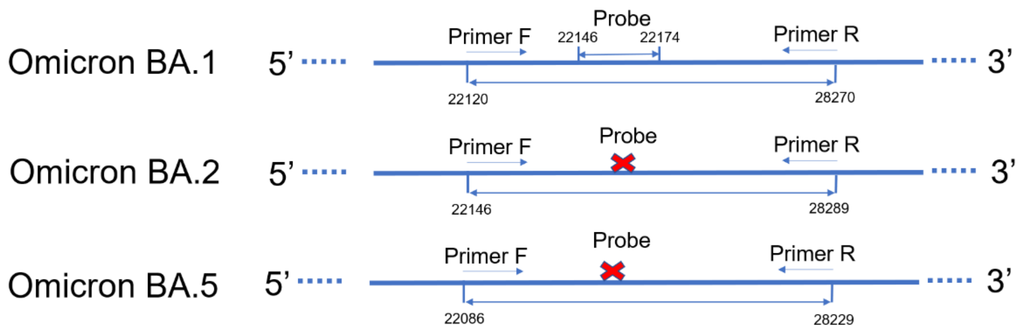

Figure S1. Primer and probe illustration in Omicron Variants

Note: Sequences of BA.1, BA.2 and NA.5 are from NCBI NC_045512.2NA.1, OX315675.1, and OP164797.1, respectively.

Supplementary Materials

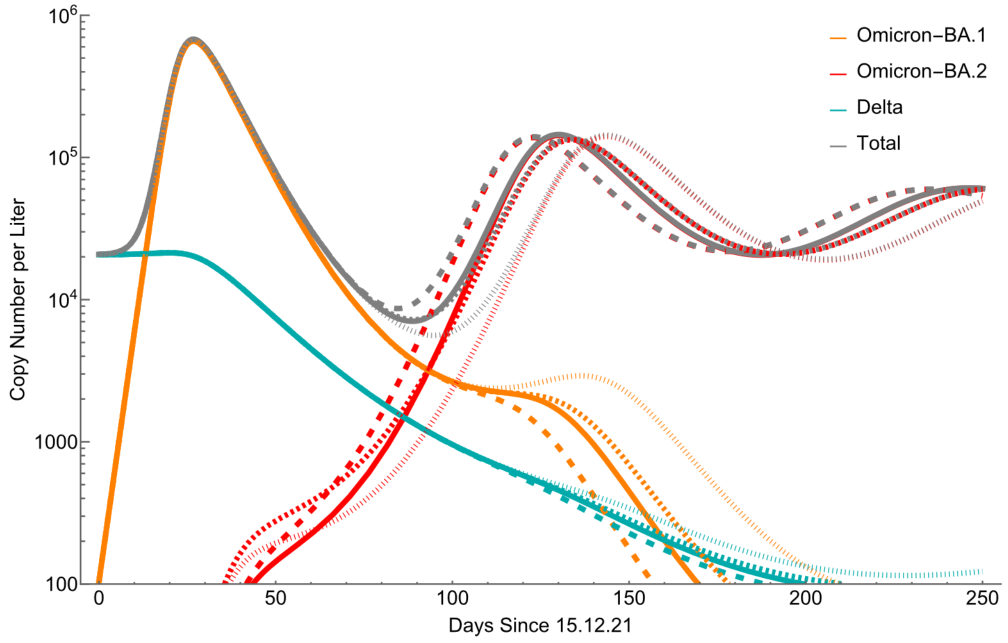

Figure S2. Sensitivity analysis to the basic reproduction number of BA.2, $R_0^{(B)}$: long dashed lines – $R_0^{(B)} = 1.6\, R_0^{(O)}$, full lines – $R_0^{(B)} = 1.5\, R_0^{(O)}$ (same as for Figure 2 in the main text), short dashed lines –$R_0^{(B)} = 1.4\, R_0^{(O)}$, shorter dashed ("dotted") lines – $R_0^{(B)} = 1.3.\, R_0^{(O)}$. All other parameters are the same as reported in Figure 2 in the main text. Weak effects are observed for BA.1 and BA.2, and negligible effects for Delta.

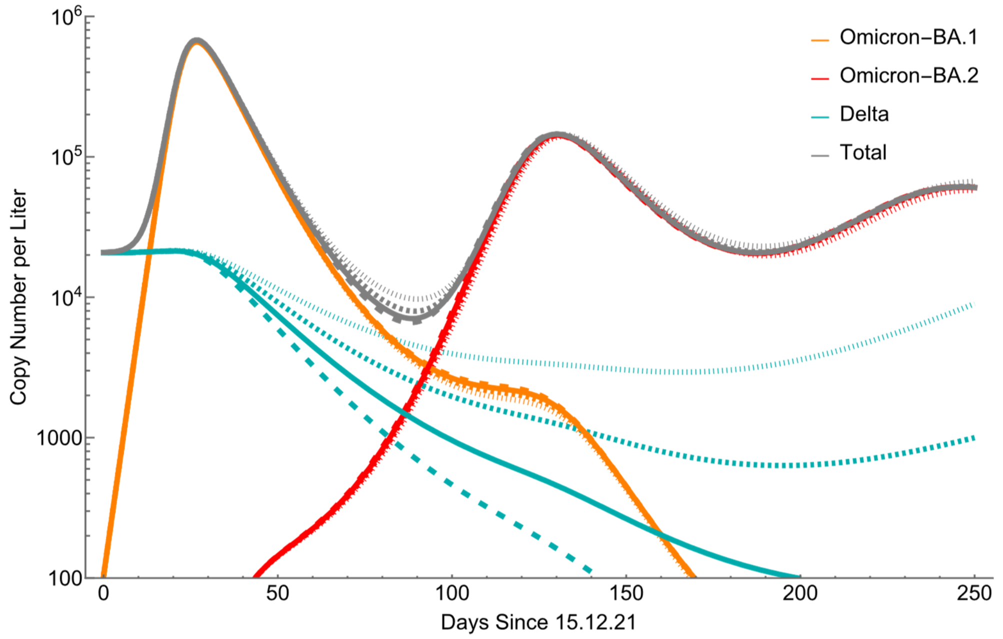

Figure S3. Sensitivity analysis to the Delta—BA.1 and Delta—BA.2 pairs cross-immunity parameters q_{DO}=q_{DB}, relative to q_{OD}=q_{BD}: long dashed lines – $q_{DO} = 0.7\, q_{OD}$, full lines – $q_{DO} = 0.6\, q_{OD}$ (same as for figure 2 in the main text), short dashed lines – $q_{DO} = 0.5\, q_{OD}$, shorter dashed ("dotted") lines – $q_{DO} = 0.4\, q_{OD}$. All other parameters are the same as reported in figure 2 in the main text. Negligible effects are observed for BA.1 and BA.2, and strong effects for Delta.

Supplementary Materials

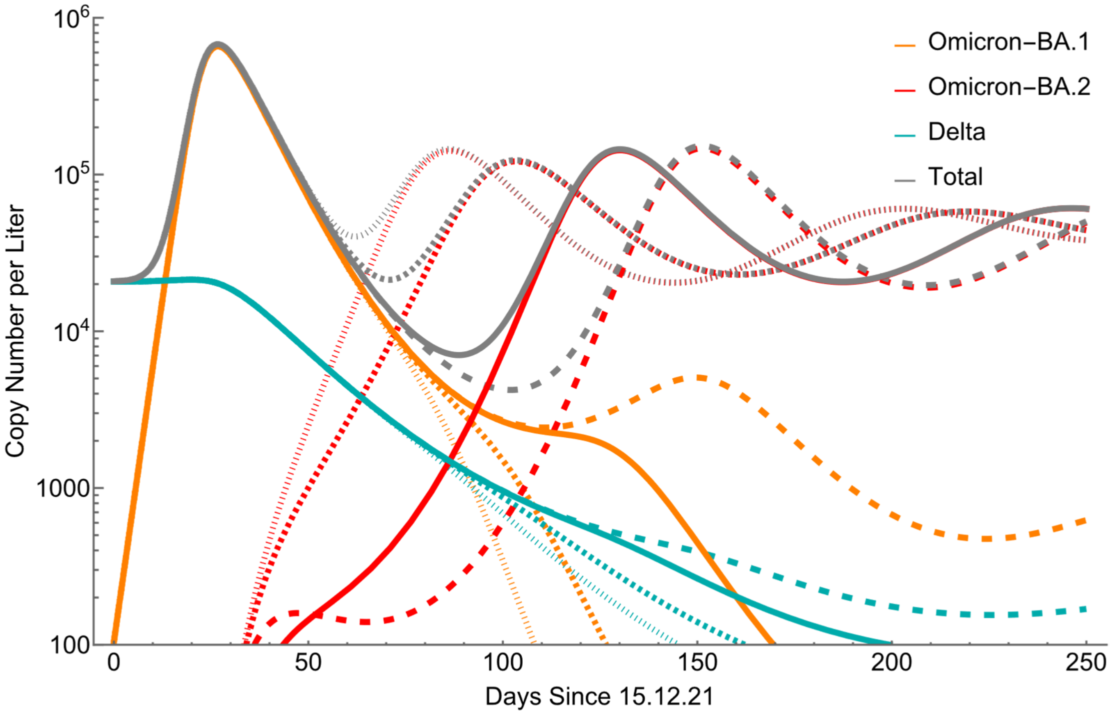

Figure S4. Sensitivity analysis to the BA.1—BA.2 pair cross-immunity parameter q_{BO}, relative to $q_{OB} = 0.8$: long dashed lines – $q_{BO} = 1.175\, q_{OB} = 0.94$, full lines – $q_{BO} = 1.0875 \times q_{OB} = 0.87$ (same as for figure 2 in the main text), short dashed lines – $q_{BO} = q_{OB} = 0.8$, shorter dashed ("dotted") lines – $q_{BO} = 0.9125\, q_{OB} = 0.73$. All other parameters are the same as reported in figure 2 in the main text. Strong effects are observed for BA.1 and BA.2, and weaker effects for Delta. In particular, the timing of the BA.2 wave is very sensitive to this value, influencing the shoulder/peak in BA.1.
